# Immunization of sheep against *Echinococcus granulosus* with protoscolex tegumental surface antigens

**DOI:** 10.14202/vetworld.2017.854-858

**Published:** 2017-08-03

**Authors:** Manouchehr Valizadeh, Behzad Haghpanah, Alireza Badirzadeh, Elham Roointan, Shirzad Fallahi, Saber Raeghi

**Affiliations:** 1 Department of Parasitology and Mycology, School of Medicine, Shahid Beheshti University of Medical Sciences, Tehran, Iran; 2 Department of Parasitology and Mycology, School of Medicine, Isfahan University of Medical Sciences, Isfahan, Iran; 3 Department of Parasitology and Mycology, School of Medicine, Lorestan University of Medical Sciences, Khorramabad, Iran; 4 Department of Laboratory Sciences, Maragheh University of Medical Sciences, Maragheh, Iran

**Keywords:** cystic echinococcosis, *Echinococcus granulosus*, immunization, Iran, Protoscolex tegumental surface antigens, sheep

## Abstract

**Aim:**

Cystic echinococcosis (CE) has potential economic effects to both animal products and human health. A vaccine to protect livestock against CE can be effective in reducing economic costs and increasing the livestock products. Protoscolex tegumental surface antigens (PSTSA) used to induce the production of specific antibodies against *Echinococcus granulosus* in sheep. The tegumental antigens were extracted from viable protoscolices by solubilization in sterile phosphate-buffered saline containing decanoyl-N-methylglucamine.

**Materials and Methods:**

Ten lambs which were infected with CE (positive control), 10 negative control, and 10 test groups of sheep were included in the study. 300 µg emulsion of purified-PSTSA was injected intramuscularly in a two-step immunization on the first and 30 days. Sera were collected immediately before immunization and 6 times with 10-day intervals until 60 days post immunization. Thereafter, the sera were tested for antibodies by indirect hemagglutination test in microtiter plate.

**Results:**

After two immunizations, all the infected animals in test group showed substantial increases in antibody titer. Statistical analysis showed a significant difference between the titer obtained in the test and negative control groups in both phases of immunization (p<0.05).

**Conclusion:**

The results showed that the PSTSA is a promising immunogenic compound for immunization of sheep against CE.

## Introduction

*Echinococcus* *granulosus* is a small cyclophyllidean tapeworm (3-5 mm long) with an indirect life cycle. Wild or domestic canids are common definitive hosts, and herbivorous or omnivorous mammals serve as intermediate hosts. Humans and livestock become infected through eating infective eggs excreted with dog feces [1,2]. Cystic echinococcosis (CE) is endemic in all inhabited continents of the world especially in regions of South America, North Africa, China, and the Middle East [3]. CE has potentially economic effects to both animal products and human health [4-6]. It is estimated that human burden of disease is 1,009,662 (95% confidence interval [CI] 862,119-1,175,654) disability-associated life years or US $763,980,979 (95% CI $676,048,731-$857,982,275) and annual livestock production loss of at least US $141,605,195 (95% CI $101,011,553-$183,422,465) and possibly up to US $2,190,132,464 (95% CI $1,572,373,055-$2,951,409,989) [4]. Iran is an important endemic area for CE. It is estimated that the overall annual cost of CE in Iran is US$232.3 million (95% CI US $103.1-397.8 million), and the annual cost associated with CE in livestock was estimated at US $132 million (95% CI US $61.8-246.5 million) [7]. Therefore, it is necessary to increase monitoring and global control of CE that could lead to a decline in human infections and also in economic loss of animals.

A vaccine to protect herbivorous livestock against CE can be effective in reducing economic costs and increasing of livestock products [8]. The EG95 vaccine against CE has proven to be highly effective [9]. Furthermore, it is established that antibody has an important role against infection with the eggs of *E. granulosus* in sheep and rodents [10-14]. However, the EG95 vaccine is not available or is very expensive in many developing countries and used only in some of the leading countries to control the infection. Therefore, it is necessary to use simpler and more convenient methods to control the CE in developing countries including Iran.

The most important intermediate host for CE, sheep, is the main source of meat in many countries in the world. Furthermore, they have substantial potential to transmission of *E. granulosus*. Hence, it is necessary to prevent their infection, both from an economic viewpoint, and also because they can be as a significant reservoir to canine and human infection. This paper describes the use of the PSTSA to induce the production of specific antibodies in sheep against infection with *E. granulosus*.

## Materials and Methods

### Ethical approval

All animal experiments were performed with the approval of the Institutional Animal Care and use Committee of the Shahis Beheshti University of Medical Science, Tehran, Iran.

### Antigen preparation

Forty hyaline hydatid cysts were collected from livers and lungs of Iranian sheep breed of the Zandi from the slaughterhouse of Tehran, the capital of Iran. All the sheeps were slaughtered up to 24 h previously. Hydatid fluid was aspirated in sterile conditions using a 10 ml syringe with a 22-gauge needle. Obtained fluids of the visible cyst were collected separately from each sample in a glass beaker by microscopic examination to determine fertile or sterile cysts at this stage. Cysts without protoscolices (PSC) and considered as sterile cysts. The PSC was allowed to sediment for 15 min, then sedimented PSC was washed three times with 100 volumes of 350 U/ml penicillin G, 250 pg/ml streptomycin in sterile phosphate-buffered saline (PBS, pH=7.4).

To eradicate the cyst membrane debris, suspended in 0.05% agar [15,16] and PSC was examined using a microscope with a magnification of 40. If PSC is alive, the eosin cannot enter to protoscolice and the live protoscolice has the green color while the dead one, absorb the color, and therefore appear red. Viability of the PSC was examined both by flame cell activity [17] and supravital staining of PSC using 0.1% eosin that the viability rates were 90-94%. Tegumental Ag was extracted from viable PSC by solubilization in sterile PBS containing decanoyl-N-methylglucamine (Mega10, Sigma, USA) as described by Carol *et al*. [18]. Protein concentrations were measured using the Biuret protein assay, and the absorbance was measured at 540 nm after 5 min of incubation at room temperature. Finally, the extracted PSTSA was kept at −70°C until used.

### Experimental animals

As positive control group, 10 CE infected lambs (Zandi breed) about 1-2 years old were obtained from slaughterhouse of Tehran, Iran. Ten negative control sheep and 10 test group sheep (free from hydatid infection) that were identical in age, sex, and breed were also obtained from the same slaughterhouse. In each three experimental group, before injection of antigen for immunization, venous blood samples were collected and then levels of *E. granulosus*-specific IgG antibodies were tested by indirect hemagglutination test (IHA) in microtiter plate.

### Immunization studies

Immunization studies were carried out in 10 Zandi sheep, which were immunized with emulsion of 300 µg purified PSTSA and equal volumes of Freund’s incomplete adjuvant via injection into the posterior thigh muscles of each hind leg. Serum samples were collected immediately before immunization and 3 times with 10-day intervals until 30 days post immunization (DPI). At 30 DPI all animals were immunized with the same volumes of PSTSA and Freund’s incomplete adjuvant (300 µg) as the above-mentioned method. The serum samples were collected at 3 times with 10-day intervals for 30 days after the second immunization with antigen. Thereafter, the sera were tested for antibodies by IHA in microtiter plate. The immunization procedure was as previously described by Patton *et al*. [19]. Titers of ≥1:128 and <1:128 were considered positive and negative, respectively.

### Statistical analysis

Statistical analysis was performed using the statistical tests of ANOVA, HSD Tukey and Chi-square to estimate the mean of IgG titer in the test group sheep. A significance level p≤0.05 was defined statistically significant.

## Results

**Figure-1:**
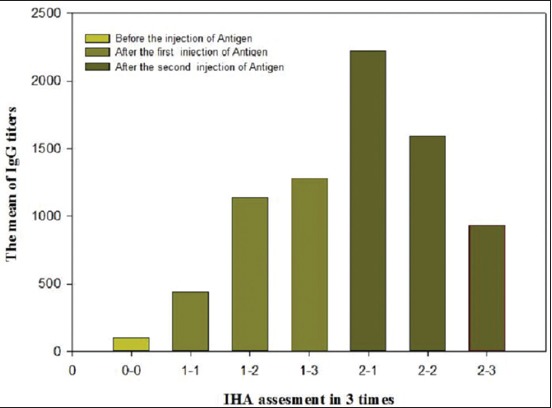
Results of injections in test group. The level of antibody was increased significantly (p≤0.05).

**Figure-2:**
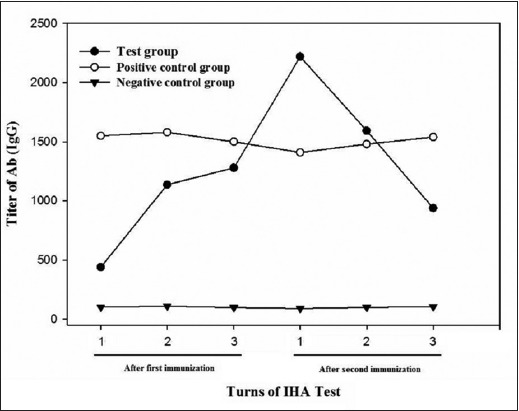
The comparative results of serological assays with indirect hemagglutination method.

The results of IHA antibody titer in immunized sheep and control groups with PSTSA antigens are presented in Table 1. In the test group, after the first immunization, all of the infected animals showed increases in antibody titer in IHA. This increase was substantial in 8 of the 10 infected animals. In second immunization phase, all of the infected animals showed substantial increases in the antibody titer obtained by IHA. In both phases of immunization, statistical analysis by ANOVA test showed that the antibody titers obtained with IHA in each three sampling time were significantly different (p<0.05) (Figure-1). Moreover, in both phases of immunization, Tukey HSD test showed that there was a significant difference between obtained titers in the test and negative control groups (p<0.05), but these values were not significant between test and positive control groups (p>0.05) (Figure-2). Statistical analysis by Chi-square test showed that the antibody titers obtained by IHA are significantly different in each 6 times after immunization (p<0.05).

**Table-1:** Indirect hemagglutination (IHA) antibody titers in sheep immunized with PSTSA and control groups.

Sheep No.	Pre-immunization titter	Control groups			Test group			Positive	Negative
	
		After first immunization (days)			After second immunization (days)				
	
		10	20	30	10	20	30		
1	1:128	1:256	1:1024	1:1024	1:1024	1:1024	1:512	1:2048	1:128
2	1:128	1:512	1:512	1:512	1:2048	1:1024	1:512	1:4096	1:128
3	1:64	1:512	1:2048	1:2048	1:4096	1:2048	1:2048	1:256	1:128
4	1:64	1:512	1:4096	1:4096	1:4096	1:2048	1:2048	1:1024	1:128
5	1:64	1:128	1:256	1:256	1:512	1:2048	1:512	1:4096	1:128
6	1:128	1:512	1:512	1:1024	1:2048	1:2048	1:1024	1:512	1:64
7	1:128	1:256	1:256	1:512	1:1024	1:1024	1:256	1:256	1:64
8	1:64	1:512	1:512	1:1024	1:2048	1:2048	1:1024	1:2048	1:64
9	1:128	1:512	1:1024	1:1024	1:2048	1:1024	1:512	1:512	1:64
10	1:128	1:512	1:1024	1:1024	1:2048	1:2048	1:512	1:512	1:128
Mean	1:102	1:440	1:1138	1:1280	1:2219	1:1593	1:939	1:539	1:102

## Discussion

The public health significance of CE has stimulated a sustained period of investigation of immunity to *E. granulosus*, particularly in sheep which are the most common intermediate host involved in transmission of the parasite [20]. Immunization of livestock intermediate host animals could have an important effect in control of the disease both from an economic aspect and also their significant contribution in canine infections and so to accidental infections of human [1,3,4,21]. A primary infection with oncospheres usually can induce total or a light degree of protection against a subsequent challenge. The created immunity to reinfection after a primary infection through either oral administration of *E. granulosus* eggs [22] and/or the parenteral injection of activated oncospheres or eggs had been reported [23]. In other words, natural immunity can be stimulated in intermediate host as the result of primary infection [13,24,25]. Some research demonstrated the potential for successful transfer of immunity against *E. granulosus* to lambs via colostrum; this study showed that exposed ewes to three oral infections with *E. granulosus* eggs, as well as immunized ewes with *E. granulosus* oncospheral antigens, transferred a remarkable level of immunity to lambs through colostrum. However, the natural immunity is often concomitant with some degrees of injury to the internal organs including liver, lung, heart, and spleen as well as economics detriments [26].

Create a long-life, strength, and effective immune response against CE both in veterinary aspect and human health has always been desirable. The role of antibody in concomitant immunity against *E. granulosus* always been debated. In this study, the prepared PSTSA was used to induce an effective immunity against the CE infection in the sheep. After two-phase of intramuscular immunization all of 10 infected animals showed substantial increases in IHA titer of specific antibodies. In the three sampling times, the increased level of specific Abs titer was significantly different (Figure-1). This finding indicates that PSTSA is an immunogenic and reactant antigen of PSC of *E. granulosus* which could be applied for effective immunization of animals including sheep against CE. There was a significant difference in the antibody titer obtained with IHA between the test and control groups in all three sampling time (p<0.05). The mean titer of antibody obtained by IHA in the test group before two stages of immunization was very lower than its amount at the 30th day of the first and second immunization (1:102, 1:1280 and 1:1939 respectively). The significant difference between obtained titers of antibody in the test and negative control groups indicate that immunization of the test group with PSTSA and incomplete Freund’s adjuvant produce a detectable CE-specific antibody response which could be protective in sheep against afterward infections.

The successfully developed methods for production of host-protective antigens from *Taenia* species to prepare excretory/secretory products from vitro culture supernatants, in which activated *E. granulosus* oncospheres had been maintained, were used before [27]. Two immunizations of lambs with these oncospheral antigens protected them against a subsequent oral challenge infection with *E. granulosus* eggs. Whereas in all control sheep numerous hydatid cysts were created as a result of challenge infection, all of the seven or eight vaccinated sheep were fully protected by immunization. Heath *et al*. [11] reported that solubilized inclusion bodies containing EG95 as a glutathione S-transferase fusion protein together with Quil A, subcutaneously induce protection in sheep and goats against *E. granulosus* infection which lasts for at least 12 months. In our study, the antibody titer obtained by IHA in each six sampling times after immunization was significantly different and with increasing the DPI, the antibody titer was also increased significantly (p<0.05).

Results of this study showed that the PSTSA of hydatid cyst beside the incomplete Freund’s adjuvant if implicated in two or three times of immunization is an effective and immunogenic compound for immunization of sheep against CE. One of the limitations of this study was the lake of determination the lasting time of protection in created immunity.

## Conclusion

The present manuscript is an attempt to raise antibody against the whole homogenate of protoscoleces, but the nature and immune protection activity of the antibody need to be tested in the other works.

## Authors’ Contributions

MV and BH, SR together have designed, planned and conducted this research. AB, ER and SF, assisted in the execution. All authors have read and approved the final version of the manuscript.
